# Supplementary material for: L-Theanine Prolongs the Lifespan by Activating Multiple Molecular Pathways in Ultraviolet C-Exposed Caenorhabditis elegans
Source: Molecules. 2024 Jun 6;29(11):2691. doi: 10.3390/molecules29112691 (PMC11173996; doi:10.3390/molecules29112691)
Supplement: Supplementary file 1 [file molecules-29-02691-s001.zip › molecules-2970779-supplementary.pdf]

Supplementary Materials: Table S1 List of primers used for the quantitative real-time reverse transcription-polymerase chain reaction.

Gene name	Primer sequence
bec-1	Fw (5′TGATCTCTGCTGACAAGGCTT3′)
	Rv (5′CCGACCTTGAATCCAGTTGG3′)
lgg-1	Fw (5′GCACCAAAGTCAAAGCTCCA3′)
	Rv (5′CCTCGTGATGGTCCTGGTAG3·)
atg-18	Fw (5·TGGGGCACAAAGATGGCTA3·)
	Rv (5·CCAAGATGTGTAAGATTTTCGCC3·)
dct-1	Fw (5′ATCGCACAATCTCCTCACGT3′)
	Rv (5′GGACAGTCTTTGGAGGTGTATT3′)
hsp-60	Fw (5′GGGGAAGCCCAAAGATCACA3′)
	Rv (5′TCCAGCCTCCTCATTAGCCT3′)
cox-4	Fw (5′GCCCCAATTCGCGCCAAGGA3′)
	Rv (5′AGGTTGGCGGCAGTTCTGGG3′)
cts-1	Fw (5′CTCGACAACTTCCCAGATAACC3′)
	Rv (5′GGTACAGGTTGCGATAGATGATAGC3′)
pyc-1	Fw (5′TCCAACTACTCCTCTTGCTACTGAC3′)
	Rv (5′GTGATCATACATCCTGGTCTACTGC3′)
hxk-1	Fw (5′GTGCGACGAGTACTTTCTCAACTG3′)
	Rv (5′CTAGAGATGACGTCACACACTTCTC3′)
tba-1	Fw (5′TGATCTCTGCTGACAAGGCTT3′)
	Rv (5′CCGACCTTGAATCCAGTTGG3′)

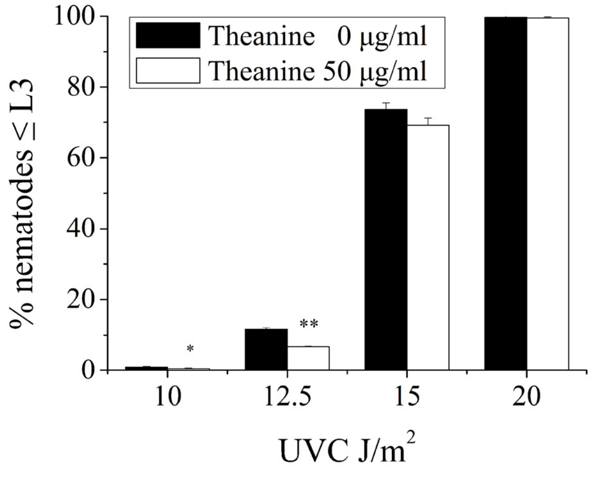

Figure S1. Effect of combination of various concentrations of L-theanine treatment and different doses of UVC irradiation on L3 arrest. The most obvious reduction of L3 arrest was in the combination of UVC (12.5 J/m^2) + L-theanine (50 μg/ml). Results are means ± SD (n = 5, t test, * $P < 0.05$, ** $P < 0.01$).

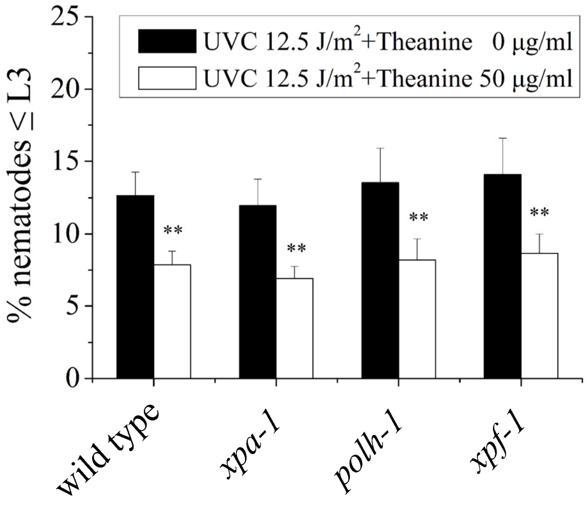

Figure S2. Mutations in NER gene (*xpa-1, polh-1*, and *xpf-1*) did not affect L3 arrest in UVC-exposed *C. elegans* treated by L-theanine. Results are means ± SD (n = 5, t test, ** $P < 0.01$).

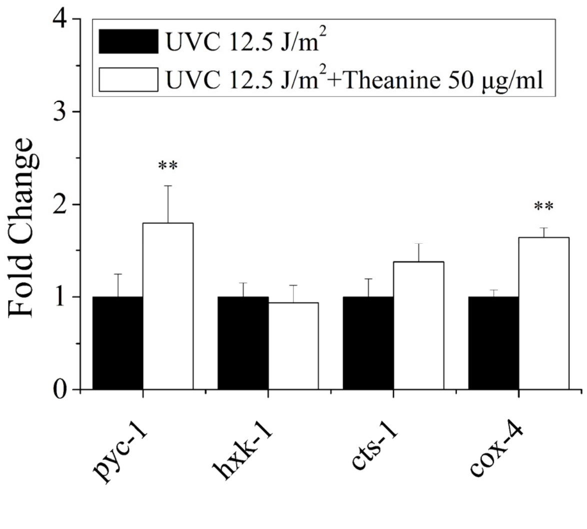

Figure S3. Effect of L-theanine treatment on the relative expression of mitochondrial energy metabolism related genes in UVC-exposed *C. elegans.* L-theanine treatment did not affect *hxk-1*(glycolysis) *and cts-1* (TCA cycle) mRNA levels in UVC-exposed *C. elegans* treated by L-theanine, but up-regulated *pyc-1* (TCA cycle) and *cox-4* (mitochondrial respiratory chain*)* expression. Results are means ± SD (n = 5, t test, ** $P < 0.01$).
